# Effect of balance training with Pro-kin System on balance in patients with white matter lesions

**DOI:** 10.1097/MD.0000000000009057

**Published:** 2017-12-22

**Authors:** Hong You, Hongxia Zhang, Jia Liu, Tingting Han, Min Zhang, Weijing Zhao, Shangrong Jiang

**Affiliations:** Sino-French Department of Neurological Rehabilitation, Gansu Provincial Hospital, Lanzhou, P.R. China.

**Keywords:** balance training, visual feedback, white matter lesions

## Abstract

Patients with white matter lesions (WMLs) often present with problems of balance. The aim of this study was to verify the effects of combined Pro-kin system and conventional balance training to improve balance ability in WMLs patients.

This is a randomized controlled study, and 40 participants were divided into 2 groups: the intervention group (n=18) received Pro-kin system with additional conventional balance training for 20 minutes per session, 5 times a week, for 2 weeks. The control group (n = 19) received only conventional balance training. Outcome measures were examined before and after the 2 weeks intervention using the Berg Balance Scale (BBS), Timed Up and Go (TUG) test, and Pro-kin system.

After completion of the 2 weeks intervention, BBS, TUG, and Pro-kin system results significantly improved in the intervention group (*P* < .05). In the control group, BBS and Pro-kin system results significantly improved (*P* < .05). Changes in all outcomes but the ellipse area with eye closed (*P* < .05) were significantly higher in the intervention group than in the control group.

The combination of Pro-kin system and conventional balance training is a potentially valuable treatment for patients with WMLs.

## 1 Introduction

Cerebral white matter lesions (WMLs) is frequently seen on brain neuroimaging in older people, which appear as low attenuation areas on CT and as areas with high signal on T2-weighted or FLAIR MRI.^[1]^ The pathology is that change of the white matter myelin in the cerebral subcortical, paraventricular, and centrum ovale.^[2]^ WMLs become increasingly common with age, which are associated with balance and gait dysfunction and falls in the elderly,^[3]^ and the impairment of sensory integration has been suggested to influence balance control in WMLs.^[4]^

Static and dynamic balance are essential functions of the human body that affect activities of daily living.^[5]^ Laufer et al^[6]^ demonstrated that the reduced ability to control balance has been associated with ambulatory dysfunction and an increased risk of falls. Therefore, static and dynamic balance training programs are an important part of rehabilitation in WMLs patients. In fact, various therapeutic methods have been used for the improvement of balance, such as core strength exercises,^[7]^ visual feedback training,^[8]^ and task-related training.^[9]^ Among the various therapeutic methods, visual feedback training is effective for improvement of balance.^[10]^ Meanwhile, the use of visual feedback training increases patient motivation, interestingness, and individualizes exercise difficulty according to a patient's current status.^[11]^

**Figure 1:**
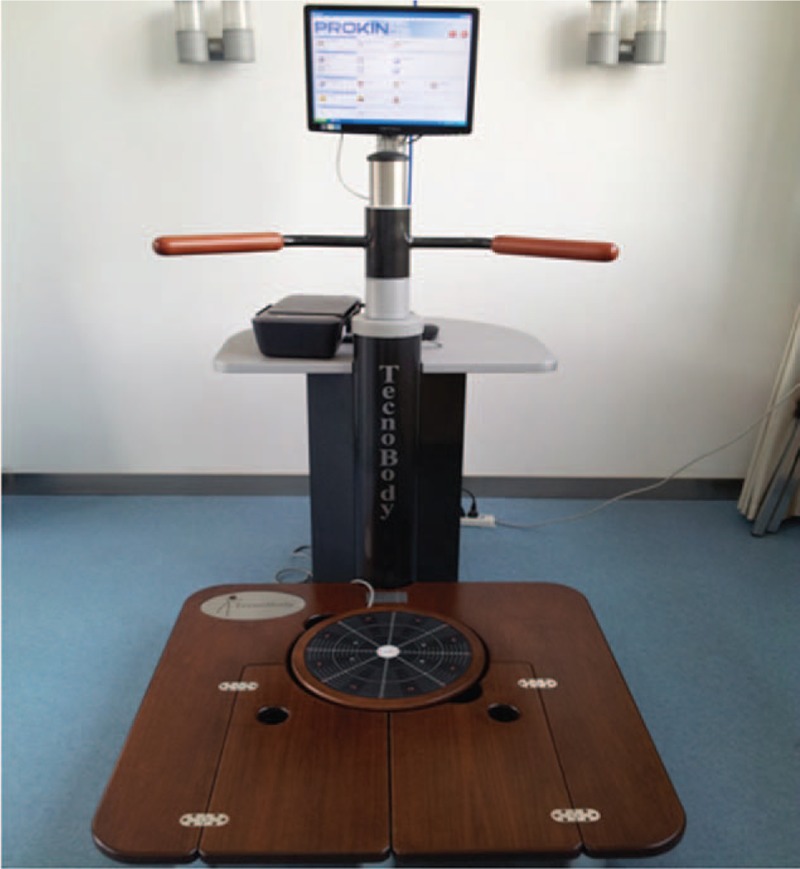
Pro-kin system.

The force platform with the visual feedback system is normally designed to provide visual representation and clues of a subject's real time center of pressure (CoP) accurately,^[12]^ which has often been used for different populations, such as stroke,^[13]^ multiple sclerosis,^[14]^ diabetic neuropathy,^[15]^ and mild traumatic brain injury.^[16]^ In this study, the Pro-kin system (Fig. 1) is a new type of visual feedback instrument, which equipped with a force platform and computer. Frazzitta et al^[17]^ demonstrated that treatment with the Pro-kin system improve balance and gait training in individuals with Parkinson's disease; however, the use of visual feedback with the Pro-kin system for balance training has not been studied in patients with balance dysfunction such as WMLs.

Therefore, the purpose of this study was to investigate the effects of visual feedback training using the Pro-kin system on balance and mobility function in patients with WMLs.

## 2 Subjects and methods

### 2.1 Study participants

The study group included 40 participants that had a radiological diagnosis of WMLs,^[2]^ who were patients of Sino-French Department of Neurological Rehabilitation of Gansu Provincial Hospital from Jun 2015 to January 2016. All participants aged 50 to 80 years were no history of leg injuries or other diseases associated with balance impairments, and Berg Balance Scale (BBS < 56), mini-mental state examination (MMSE > 22). Participants were excluded if they had any musculoskeletal, cardiovascular, or respiratory system impairments or other accompanying ailments. Individuals who participated in less than 80% of the exercise program and those who were unable to perform follow-up tests were also excluded from the final analyses.

All participants were randomly divided into an intervention group (n = 20) or control group (n = 20). However, 2 patients in the intervention group and 1 patient in the control group were excluded from the analysis because of participation in less than 80% and follow-up tests unfinished. The study included in total 37 participants: 18 in the intervention group and 19 in the control group. All subjects provided written informed consent.

### 2.2 Data collection

The balance ability was calculated by the Berg Balance Scale (BBS)^[18]^ and the Timed Up and Go test (TUG).^[19]^ The BBS is a clinical functional measurement of balance impairment, consists of 14 items of increasing difficulty, which are scored on a 5-point ordinal scale (0–4), the maximum possible score is 56, and higher scores indicate better balance. In this study, the TUG was also performed to assess the balance of the subjects, it records the time required for subjects to stand up from a chair, walk 3 m, turn around, return, and sit down again. The 2 tests were repeated twice and recorded the mean scores as the result.

This study has also used the Pro-kin system (PK254, TenoBody s.r.l. Bergamo, Italy) to assess balance, which is based on the assessment of postural sway using the force platform from movements of the center of pressure (CoP). Subjects stood comfortably corresponding position on the platform; they were instructed to look straight ahead at a screen surface placed and to keep arms at their sides during the stances in a normal forward-facing position, with eyes focused on a stationary target. Each participant performed 2 standing tests, in the open eyes (OE) and closed eyes (CE), respectively, each test lasting 30 seconds. Four different outcome variables were calculated in 2 conditions; these variables are: perimeter (measured in mm) and the ellipse area (measured in mm^2^). The test was performed twice, and the mean score was recorded.

### 2.3 Procedures

All participants received conventional balance training, which consisted of 5 practices for 20 minutes per session, 5 times a week, for 2 weeks, as follows: (1) standing on 1 leg for 5 seconds; (2) standing in front of the mirror, therapists push patients from different directions; (3) weight shifting forward, backward, sideward, and diagonally with eyes opened and eyes closed; (4) passing balls to therapist arranged in a circle, throwing and catching a ball; (5) walking in a straight line.

**Figure 2:**
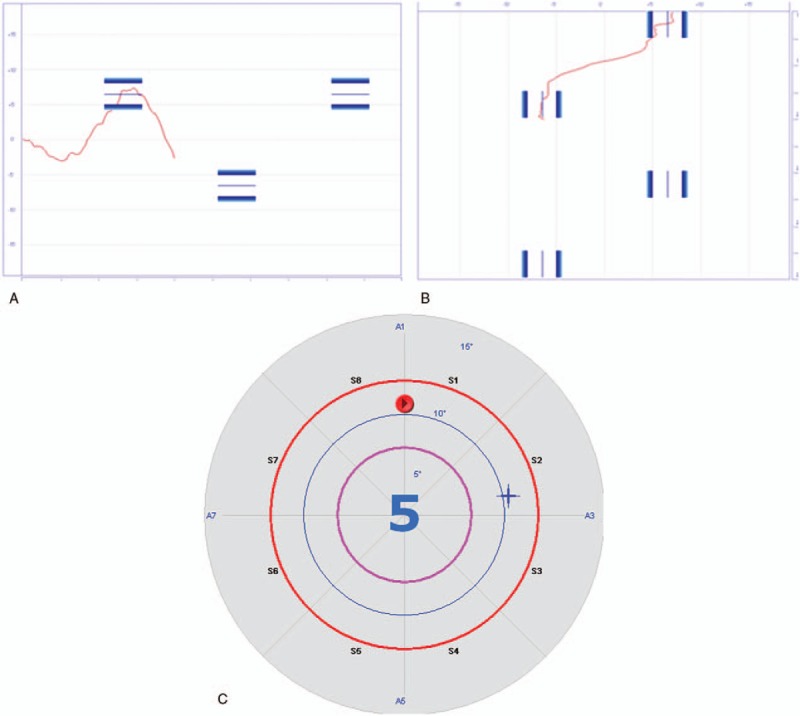
Training using the Pro-kin system by various ways. (A) Forward and backward, (B) sideward, (C) circular motion.

**Figure 3:**
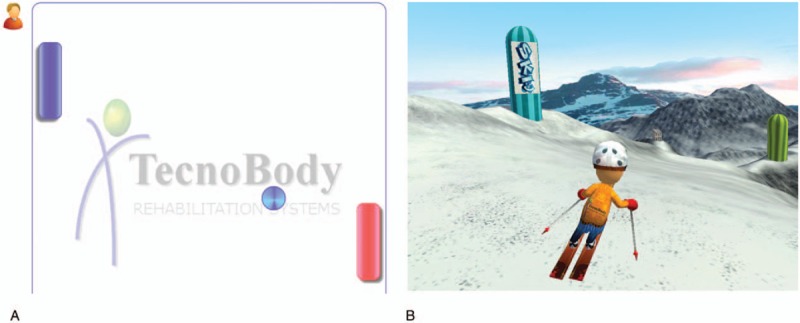
Screenshot of the game. (A) Table tennis, (B) ski.

However, all the subjects in the intervention group performed balance training using the Pro-kin system in addition to the conventional training, for 20 minutes per session, 5 times a week, for 2 weeks. Using visual feedback sensitive to the displacement of the center of pressure (CoP), patients had to move their CoP pass the specified area, by various ways including forward, backward, sideward, and circular motion (Fig. 2), and performed 2 games including table tennis and ski (Fig. 3).

### 2.4 Statistical analysis

All statistical tests were performed with SPSS version 17.0. The values were compared between groups using the independent *t*-test, and before and after the intervention by the paired *t*-test. Differences in categorical variables were analyzed using the χ^2^ test. For all tests, *P* < .05 was considered to indicate statistical significance.

## 3 Results

The general characteristics of the participants, age, gender, height, weight, BMI, scope lesion are described in Table 1; there were no significant differences between groups. The BBS, TUG before and after training and changes in scores are shown in Table 2. Pro-kin system results before and after training and changes in all outcomes were calculated in Table 3. There was no significant difference between groups before the training; however, compared with the control group, BBS (*P* < .001), TUG (*P* = .001), and Pro-kin balance system results were significantly improved after training in the intervention group. Comparing the results before and after the training in each group, TUG and perimeter with CE were no significant differences in the control group. However, in the intervention group, BBS (<.001), TUG (*P* = .001), perimeter with OE (*P* < .001), the ellipse area with OE (*P* < .001), perimeter with CE (*P* < .001), and the ellipse area with CE (*P* = .006) were significantly improved after the training. For better evaluation of the findings between groups, the changes in all outcomes before and after were calculated. This study observed that the intervention group had higher changes in all outcomes but the ellipse area with CE.

**Table 1:**
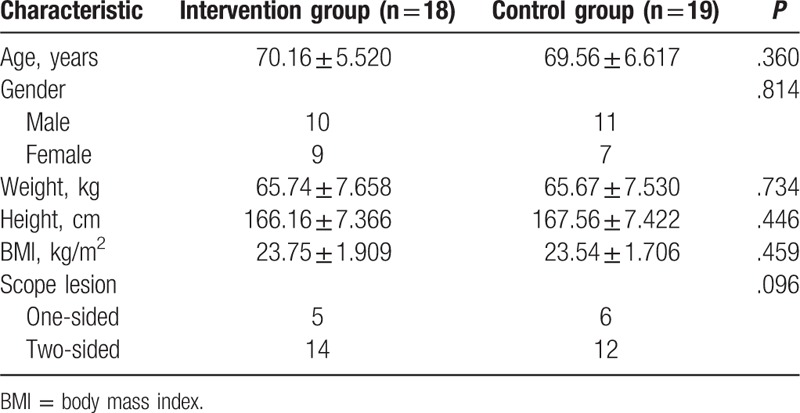
Characteristics of the participants.

**Table 2:**
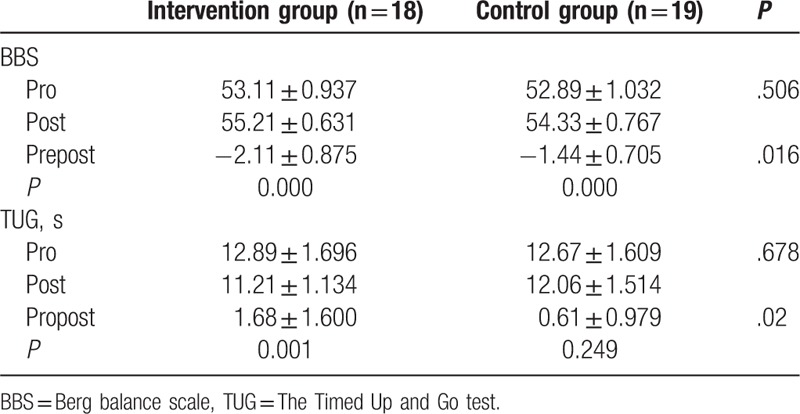
BBS, TUG before and after training.

**Table 3:**
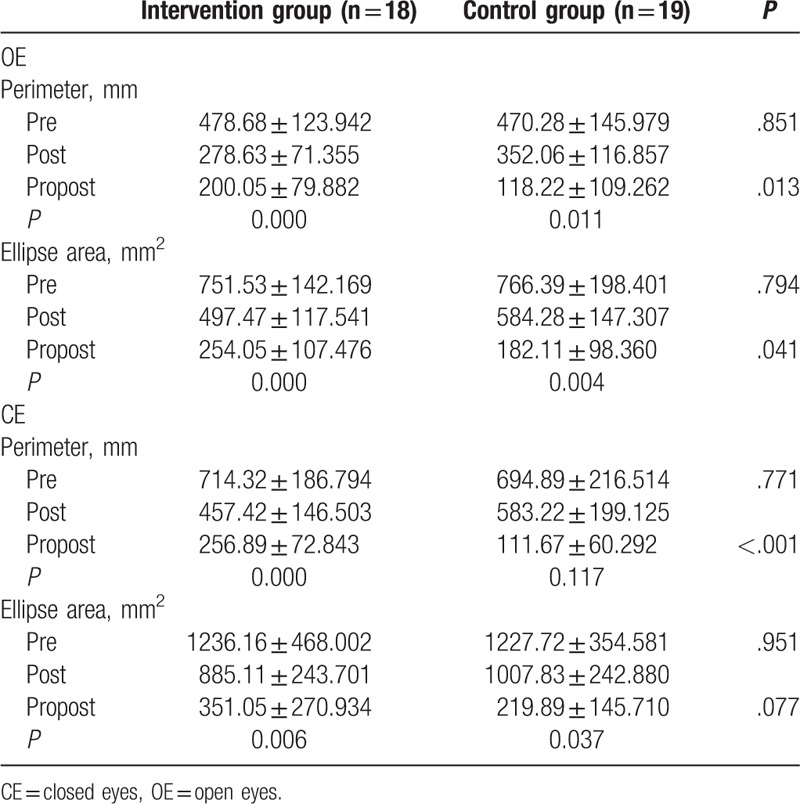
Pro-kin system results before and after training.

## 4 Discussion

A number of investigators have used visual feedback to improve standing posture and balance. Sihvonen et al^[20]^ showed that visual feedback based balance training reduced the incidence of falls among frail older women. Research has also shown that using visual feedback is an effective instructional method for enhancing balance ability in a poststroke population.^[10,12,21]^ Others previous studies have investigated the efficacy of visual feedback training in improving balance in different populations, reported that in multiple sclerosis,^[14]^ diabetic neuropathy,^[15]^ and mild traumatic brain injury^[16]^ when compared with conventional standing balance training, but there is little evidence regarding the effectiveness in WMLs patients.

This study showed for the first time that balance training using visual feedback with additional Pro-kin system training produced a significant improvement, which was greater than conventional balance training alone in enhancing the balance ability of WMLs patients.

It has been reported that visual feedback training using a Biodex Balance Master was significantly effective for the improvement of balance in stroke patients.^[12]^ In this study, the Pro-kin system which is similar to the Biodex Balance Master but it is a force platform with a flat and regular surface fixed to 4 force-transduction systems, unlike the study of Srivastava et al,^[12]^ the groups consisted of patients with WMLs, but the results were similar, this study also suggest that using force platform feedback in addition to a conventional training in patients with WMLs is beneficial in improving balance. In this study, the BBS and TUG test were performed before and after 2 weeks of training. In comparison of the results from before and after training, the BBS were improved after the 2 weeks of training in both groups, the TUG were improved in the intervention group, but it was no statistical differences in the control group. The possible reason is that the training term was too short or the conventional balance training was sufficient to enable the patients to maximize their potential or that Patients’ lack of interest and initiative.

The Pro-kin system results were also significantly improved after 2 weeks of balance training. In the intervention group with OE, the perimeter decreased by 41.8% and the ellipse area decreased by 33.8% in the intervention group, and in the eyes closed condition, the perimeter decreased by 35.9% and the ellipse area decreased by 28.4%. In the control group with OE, the perimeter decreased by 25.1% and the ellipse area decreased by 23.8%, and in the eyes closed condition, the perimeter decreased by 16.1% and the ellipse area decreased by 17.9%. Compared with the control group, conventional balance training additional Pro-kin system was significantly improved. Actually, during the process of training, weight or posture shifting, the position and movement tracks of center of gravity can be monitored, and thus, subjects can recognize by visual feedback to adopt appropriate strategies to keep postural control as steady as possible.^[22]^ This study observed that, after treatment, perimeter and ellipse area in the intervention group were significantly lower than the control group. Similar to the previous studies using the Pro-kin system on Parkinson's Disease, the results here suggest that that using the force platform biofeedback has the effect on balance dysfunction of Parkinson's patients.^[17]^ Moreover, in this study, perimeter and ellipse area with OE were lower than that in the closed eyes, and the changes of the ellipse area with EC have no significant differences. The reason is that visual information may compensate for the loss of somatosensory function and facilitate the human motor program in the brain; thus, it would increase the effectiveness of treatment.^[23]^

There are few limitations in this study such as small sample size that is the failure to distinguish between levels of WMLs and was a short-term study lasting for 2 weeks. Although it has demonstrated that the effects of Pro-kin system, the other important limitation of this study is the lack of a follow-up period in order to evaluate the persistence of beneficial effects. Further studies are necessary to address this issue.

In conclusion, this study shows that using Pro-kin system combination with conventional training is a feasible method for balance training in WMLs patients. Compared with conventional balance training alone, this method was significantly effective in improving balance ability.

## Acknowledgments

The authors would like to thank all of the study participants.
